# The Calcium-Sensing Receptor and Integrins in Cellular Differentiation and Migration

**DOI:** 10.3389/fphys.2016.00190

**Published:** 2016-05-26

**Authors:** Sujeenthar Tharmalingam, David R. Hampson

**Affiliations:** Pharmaceutical Sciences, University of TorontoToronto, ON, Canada

**Keywords:** calcium, extracellular matrix, G-protein coupled receptor, GPRC6A, cerebellum, cerebellar granule cells, metastasis, medulloblastoma

## Abstract

The calcium-sensing receptor (CaSR) is a widely expressed homodimeric G-protein coupled receptor structurally related to the metabotropic glutamate receptors and GPRC6A. In addition to its well characterized role in maintaining calcium homeostasis and regulating parathyroid hormone release, evidence has accumulated linking the CaSR with cellular differentiation and migration, brain development, stem cell engraftment, wound healing, and tumor growth and metastasis. Elevated expression of the CaSR in aggressive metastatic tumors has been suggested as a potential novel prognostic marker for predicting metastasis, especially to bone tissue where extracellular calcium concentrations may be sufficiently high to activate the receptor. Recent evidence supports a model whereby CaSR-mediated activation of integrins promotes cellular migration. Integrins are single transmembrane spanning heterodimeric adhesion receptors that mediate cell migration by binding to extracellular matrix proteins. The CaSR has been shown to form signaling complexes with the integrins to facilitate both the movement and differentiation of cells, such as neurons during normal brain development and tumor cells under pathological circumstances. Thus, CaSR/integrin complexes may function as a universal cell migration or homing complex. Manipulation of this complex may be of potential interest for treating metastatic cancers, and for developmental disorders pertaining to aberrant neuronal migration.

## Introduction—general features of CaSR structure, activation, and expression

The CaSR belongs to the metabotropic glutamate receptor subclass of G-protein coupled receptors (GPCRs). In addition to the eight metabotropic glutamate receptor subtypes, other members of this relatively small GPCR subfamily include the GABA_B_ receptor, several taste receptors, and GPRC6A (reviewed in Conigrave and Hampson, 2010). Among these family members, the CaSR has particularly high homology, in terms of both protein sequence (31% amino acid identity) and gene structure, with GPRC6A (Kuang et al., 2005). Interestingly, GPRC6A is activated directly by several amino acids, the most potent of which are arginine and lysine, and is allosterically potentiated by extracellular Ca^2+^ (Kuang et al., 2005; Wellendorph et al., 2005), whereas the CaSR is activated directly by Ca^2+^ and allosterically modulated by amino acids (Conigrave et al., 2000). This similar but reciprocal pharmacological relationship is likely a reflection of the evolutionary origins of the two receptors (Kuang et al., 2006). The close relationship between these two GPCRs is further illustrated by reports that both receptors are activated by multiple endogenous ligands (see Clemmensen et al., 2014 for review). For example, in addition to amino acids, GPRC6A is also activated by testosterone (Ko et al., 2014), and some groups have reported that osteocalcin, a bone-derived peptide that participates in pancreatic beta cell function, is also an endogenous ligand (Pi et al., 2011; De Toni et al., 2014; Wei et al., 2014); although this remains controversial (see Rueda et al., 2016).

The CaSR is a large heptahelical transmembrane glycoprotein consisting of 1078 amino acids with a molecular weight of approximately 120 kilodaltons. Like other members of the metabotropic glutamate receptor family, it likely exists in the plasma membrane in a dimeric configuration (Bai et al., 1999; Hu and Spiegel, 2007). In most cases the cellular event that initiates activation of the receptor is a rise in extracellular Ca^2+^. However, in addition to Ca^2+^ ions the receptor can also be activated by a several other di- and trivalent cations and organic molecules such as the polyamines (Leach et al., 2014), some of which likely operate as additional or alternative endogenous ligands. A salient feature of this receptor is that it contains multiple Ca^2+^ binding sites within the active oligomeric complex and displays a high degree of positive cooperativity of activation (Breitwieser, 2013). In functional biochemical assays, the Hill coefficient of activation is typically 4–6 (Miedlich et al., 2002; Tharmalingam et al., 2011; Conigrave and Ward, 2013) and up to five Ca^2+^ binding sites have been predicted for the receptor (Zhang et al., 2014). The presence of multiple cation binding sites operating in a highly positive cooperative fashion translates into a state whereby small changes within a narrow concentration range of extracellular Ca^2+^ (1.1–1.3 mM) fully and rapidly activates the receptor.

The CaSR has been linked to a wide range of physiological processes. The first to be discovered and the most widely studied of these biological roles is its expression in the chief cells of the parathyroid gland and the regulation of parathyroid hormone release. The development of the drug Cinacalcet to treat hyperparathyroidism was based on the ability of the CaSR to suppress parathyroid hormone release from the parathyroid gland. Cinacalcet is a positive allosteric modulator of the CaSR. Its approval by the FDA in 2004 for the treatment of hyperparathyroidism represented the first commercial development of an allosteric modulator of a GPCR (Nemeth, 2013). The biochemical basis of Cinacalcet's efficacy in treating hyperparathyroidism with minimal side effects is likely achieved by the extremely high level of expression of the CaSR in the adult parathyroid gland. In addition to the parathyroid gland, the CaSR is also widely expressed in other tissues, and therefore its physiological functions extend beyond controlling parathyroid hormone release. These functions include suppression of vitamin D synthesis which lowers the drive for intestinal Ca^2+^ absorption, negative modulation of parathyroid hormone-induced phosphate excretion to raise the serum inorganic phosphate level, and decreased osteoclastic-dependent bone resorption (Leach et al., 2014). The CaSR's role in these processes are carried out by the activation of a variety of intracellular signaling pathways including stimulation of IP_3_ turnover and the release of intracellular Ca^2+^, the activation of ERK and Akt, and other signaling pathways. It has also become apparent that the CaSR is involved in additional processes including cellular differentiation and migration, brain development, stem cell engraftment, wound healing, and tumor growth and metastasis. This review will focus on the role of the CaSR in the differentiation and movement of cells, and the mechanisms underlying these processes in the developing CNS and in cancer. A summary of several key studies linking the CaSR with cell migration is shown in Table 1.

**Table 1:** **Summary of studies linking the CaSR and cellular migration**.

**Cells implicated in CaSR mediated cell migration**	**Intracellular signaling pathways involved in CaSR mediated cell migration**	**References**
Cerebellar granule cell precursors (GCP)	ERK2 and AKT mediated increase in plasma membrane expression of β1 integrins	Tharmalingam et al., 2016
Renal carcinoma cells	AKT, PLC, JNK, p38	Joeckel et al., 2014
Cardiac fibroblasts	PLC, IP3	Zhang et al., 2014
Rat medullary thyroid carcinoma cells	PLC, [Ca^2+^]_*i*_, ERK1/2	Tharmalingam et al., 2011
Human bronchial epithelial cells	PLC, ERK1/2	Milara et al., 2010
Monocyte-macrophage lineage cells (RAW 264.7)	(PI3K), Akt, PLC	Boudot et al., 2010
Breast cancer cells (MDA-MB-231)	ERK1/2, PLC	Saidak et al., 2009
Gonadotropin-releasing hormone neurons (GnRH)	CaSR activation promotes monocyte chemoattractant protein-1 (MCP-1) secretion which binds CC motif receptor-2	Chattopadhyay et al., 2007
Haematopoietic stem cells (HSC)	Intracellular signaling mechanism was not determined	Adams et al., 2006
Murine osteoblastic cell line (MC3T3-E1)	Intracellular signaling mechanism was not determined	Yamaguchi et al., 1998
Murine bone marrow-derived stromal cell line (ST2)	Intracellular signaling mechanism was not determined	Yamaguchi et al., 1998

## The CaSR in hematopoietic cell homing, and bone cell differentiation, and migration

During mammalian ontogeny, hematopoietic stem cells (HSCs) translocate from the fetal liver to the bone marrow where hematopoiesis occurs throughout adulthood. A characteristic of bone that likely contributes to an attractive microenvironment for stem cells is the high concentration of calcium ions at the HSC-enriched surface of the endosteum (the endosteum is the thin vascular membrane of connective tissue that lines the surface of the bony tissue that forms the medullary cavity of long bones). In fact, the bone endosteal surface has been shown to act as a niche for circulating CaSR-expressing HSCs to preferentially localize to the bone (Adams et al., 2006). HSCs prepared from mice lacking the CaSR were defective in localizing to the endosteal niche, and additionally, HSC maturation was altered as indicated by the observation that progenitor cells were present in an immature form in the circulation and spleen. Although CaSR^−∕−^ HSCs from fetal liver were normal in number and in proliferative capability, they were highly defective in localizing anatomically to the endosteal niche, a characteristic that correlated with defective adhesion of the HSCs to collagen I, an extracellular matrix protein (ECM) present in the endosteal surface (Adams et al., 2006). Together, these results indicate that the CaSR is responsible for retaining and adhering HSCs in close physical proximity to the bone endosteal surface.

Additional *in vitro* studies have shown that CaSR stimulation with Cinacalcet increased HSC growth in stromal cell co-cultures (determined using the “cobblestone area-forming cell assay” which measures progenitor cell-like and stem cell-like activities) by promoting HSC adhesion to ECM proteins such as collagen I and fibronectin (Lam et al., 2011). Moreover, co-stimulation of CXCR4 (a GPCR) and the CaSR resulted in augmented *in vivo* homing to the endosteal niche and engraftment capacity. This work suggested that modulation of the CaSR might be a viable strategy for enhancing HSC engraftment in bone marrow (Lam et al., 2011).

The role of the CaSR in HSC homing has been further established using a biodegradable composite biomaterial composed of Ca^2+^ phosphate glass/polylactic acid which was developed to mimic elevated Ca^2+^ levels surrounding the bone microenvironment (Aguirre et al., 2012). Using this biomaterial, Aguirre et al. (2012) demonstrated that bone marrow-derived HSC mobilization, differentiation, and angiogenesis occurs via CaSR activation in the presence of elevated extracellular Ca^2+^. One mechanism in which the CaSR promotes HSC homing to the bone environment is by increasing the expression of CXCR4 in the presence of elevated extracellular Ca^2+^ (Wu et al., 2009). CXCR4 is involved in leukocyte trafficking and antagonists of this receptor are being developed for the treatment of inflammatory diseases, cancer, and HIV. CXCR4 regulates homing of leucocytes, endothelial progenitors, and bone marrow cells in response to SDF-1 present in the bone endosteal niche; here, extracellular Ca^2+^ acting through CaSR activation augments SDF-1 signaling by serving as a positive regulator of CXCR4 expression to promote stem cell mobilization and homing (Wu et al., 2009).

The CaSR is expressed in both osteoclasts and osteoblasts, the cells involved with resorption of the mineralized bone matrix and cells that replace the resorbed bone, respectively (Sugimoto et al., 1993; Marie, 2010). The dynamic balance between osteoclasts and osteoblasts determines bone remodeling and serum Ca^2+^ concentrations. Bone tissue likely has elevated Ca^2+^ levels compared to other tissues. However, studies reporting actual measurements of Ca^2+^ in bone are sparse, typically use microelectrode-based measurements, and differ widely in the estimates of Ca^2+^ concentrations. An early study reported the extracellular level of Ca^2+^ in bone to be about 27 mM, and at sites of osteoclastic bone resorption the local Ca^2+^ concentration was estimated to be as high as 40 mM (Silver et al., 1988). In another analysis performed using microelectrode measurements in bone slice cultures, the extracellular Ca^2+^ level was estimated to be 2 mM at sites of osteoclast mediated bone turnover (Berger et al., 2001). However, the fact that the latter estimate was derived from tissue slices *in vitro* leaves open the question of its applicability in the intact bone. In any case, since maximum CaSR responses are typically achieved at 2–4 mM extracellular free calcium (Tharmalingam, 2014), even the lower of the two estimates for bone cited above is within the range of CaSR activation.

CaSR-expressing osteoblasts appear to utilize the CaSR as a chemoattractant receptor to sense elevated extracellular Ca^2+^ at osteoclast mediated bone resorption sites. Migration of CaSR-expressing osteoblasts to bone remodeling sites allows replacement of the missing bone during the osteoblastic phase of bone turnover (Sugimoto et al., 1993; Theman and Collins, 2009). Signaling studies demonstrate that CaSR stimulation in osteoblasts results in activation of phospholipase C (PLC), extracellular signal-regulated kinase (ERK1/2), and JNK signaling cascades. These CaSR-stimulated signaling pathways contribute to osteoblast migration, differentiation and bone remodeling (Sharan et al., 2008; Yamaguchi, 2008; Marie, 2010).

Similar to osteoblast migration, localization and homing of CaSR-expressing osteoclast precursor cells to the bone environment is important for initiating bone remodeling. Using RAW 264.7 cell line derived from murine osteoclast precursor cells, Boudot et al. (2010) demonstrated that extracellular Ca^2+^ mediated activation of the CaSR was crucial for migration of these cells in a directional manner. The phosphoinositide 3-kinase (PI3K)/Akt and PLCβ signaling pathways were identified as mediators in the migratory effect. These results suggest that the presumed extracellular Ca^2+^ gradient present in bone is an initiating factor for the homing of osteoclast precursors, and may play a role in the initiation of bone remodeling (Boudot et al., 2010).

A final example of CaSR-mediated cellular migration is illustrated by the migration of gonadotropin-releasing hormone neurons in the hypothalamus. The CaSR is expressed in primary cultures of gonadotropin-releasing hormone neurons from murine basal forebrain, and in two different gonadotropin-releasing hormone neuronal cell lines (Chattopadhyay et al., 2007). Activation of the CaSR with elevated extracellular Ca^2+^ promoted gonadotropin-releasing hormone neurons to engage in directional chemotaxis. CaSR stimulation resulted in Ca^2+^ influx through N-type calcium channels and subsequent secretion of monocyte chemoattractant protein which synergistically promoted gonadotropin-releasing hormone neuron migration toward extracellular Ca^2+^ (Chattopadhyay et al., 2007). These *in vitro* experiments were supported by studies in CaSR knockout mice where CaSR null gonadotropin-releasing hormone neurons showed approximately 27% fewer neurons in the final resting position in the preoptic area of the anterior hypothalamus compared to wild-type littermates (Chattopadhyay et al., 2007).

## The CaSR in cancer

An *in silico* analysis of GPCR gene expression profiling gleaned from microarray data sets of non-small cell lung cancer, breast cancer, prostate cancer, melanoma, gastric cancer and diffuse large B cell lymphoma, indicated that the CaSR is up-regulated in primary and metastatic cancer cells compared to normal tissue (Li et al., 2005). Over the past 15 years many studies have reported that the CaSR can act to either promote or suppress tumorgenesis and metastasis depending on the cancer type (Brennan et al., 2013). For example, in parathyroid and colon cancers, CaSR expression is significantly reduced leading to loss of the growth suppression when exposed to elevated extracellular Ca^2+^. Activation of the CaSR in these tumors has been reported to decrease cell proliferation and tumor progression (Singh et al., 2013). Thus, CaSR expression decreases the tumorigenicity of parathyroid and colon cancers. Conversely, activation of the CaSR facilitates metastasis to bone in breast, prostate, renal and various other invasive cancers (Brennan et al., 2013). In addition, increased bone resorption induced by dietary Ca^2+^ deficiency promotes breast cancer bone metastasis, while increased dietary Ca^2+^ intake has preventative effects in colon cancer and parathyroid tumor progression (Lipkin, 1999; Butler et al., 2010; Macleod, 2013).

Similar to HSC homing, several types of primary tumor cells migrate to bone tissue. Metastatic cells preferentially attach to regions of active bone turnover and remodeling (e.g., blood, femur, pelvis, ribcage, and skull). The CaSR is expressed in both normal breast ductal epithelial cells and in primary breast cancer cells. Stimulation of the CaSR has been shown to promote the migration of several metastatic bone-preferring breast cancer cell lines. Highly bone metastatic MDA-MB-231 breast cancer cells display more robust migration compared to the MCF7 and T47D breast cancer cells, which have a much lower metastatic potential *in vivo* (Liu et al., 2009). MDA-MB-231 cells are estrogen receptor-positive while MCF-7 cells are estrogen receptor-negative suggesting the role of estrogen responsive factors in the control of CaSR function. Moreover, the BT474 breast cancer cells, which do not metastasize to the bone, did not respond to CaSR stimulation. Thus, extracellular Ca^2+^ appears to be a chemoattractant for bone preferring metastatic breast cancer cells toward a Ca^2+^ rich environment, and importantly, the level of CaSR expression has been shown to correlate positively with the magnitude of breast cancer metastasis potential (Saidak et al., 2009).

Breast cancer metastasis to bone results in increased osteolytic activity at the site of invasion. In highly invasive MDA-MB-231 cells, stimulation of the CaSR induces secretion of multiple cytokines and growth factors that result in endothelial cell migration and *in vitro* angiogenesis (Hernandez-Bedolla et al., 2015). These effects were inhibited by anti-CaSR blocking monoclonal antibodies and the calcilytic (CaSR antagonist) NPS-2143. The CaSR was also shown to mediate a pro-angiogenic environment at the site of invasion by promoting the secretion of pleiotropic molecules such as GM-CSF, EGF, MDC/CCL22, FGF-4, and IGFBP2, which are characteristic chemotactic and angiogenic factors. Conversely, constitutive secretion of IL-6 and β-NGF was partially inhibited by CaSR stimulation in MDA-MB-231 cells. However, in normal mammary cells, constitutive secretion of RANTES, angiogenin, and oncostatin M was attenuated by CaSR activation, whereas secretion of IL-6 was increased. Thus, altered secretion of chemotactic and pro-angiogenic cytokines in breast cancer cells is modulated by the CaSR (Hernandez-Bedolla et al., 2015).

The CaSR also modulates breast cancer metastasis by mediating the secretion of parathyroid hormone-related peptide (PTHrP). During lactation, activation of the CaSR in normal mammary epithelial cells down-regulates PTHrP in milk and in the circulation, and increases Ca^2+^ transport into milk. However, a switch in CaSR G-protein usage during malignant transformation appears to convert this feedback loop into a PTHrP feed-forward cycle in breast cancer cells that exacerbates the growth of osteolytic skeletal metastases (Vanhouten and Wysolmerski, 2013). CaSR stimulated secretion of PTHrP from breast cancer cells promotes osteolytic activity and is increased by transforming growth factor-β released from resorbed bone. Hence, in PTHrP-secreting breast cancers that metastasize to bone, the CaSR initiates a vicious cycle in which PTHrP-induced bone resorption raises the concentration of extracellular Ca^2+^ and transforming growth factor-β within the bone microenvironment, which then act in concert to evoke further PTHrP release and worsening osteolysis (Sanders et al., 2001). In some clinical cases, humoral hypercalcemia of malignancy occurs when secretion of PTHrP by cancer cells causes hypercalcemia in the absence of skeletal metastases (Sanders et al., 2000).

In contradiction to the studies described above demonstrating CaSR expression up-regulation in breast cancer progression, another study of CaSR protein expression in 148 cases of breast cancer using tissue microarray by immunohistochemistry reported lower CaSR expression in breast cancer lesions compared with paired non-tumor tissues (Li et al., 2014). A multivariate analysis showed that CaSR was of independent prognostic significance for both overall survival and cause-specific survival of breast cancer patients; the patients with lower expression of CaSR were also significantly associated with distant metastasis-free survival. Therefore, this dataset suggested a tumor suppressor role of the CaSR in breast cancer (Li et al., 2014). In summary, most but not all of the evidence favors a role of the CaSR in facilitating breast cancer metastasis; additional confirmatory studies need to be conducted looking at levels of the CaSR in human breast cancer biopsy samples in order to validate the findings of immortalized breast cancer cell models described above.

Similar to metastatic breast cancer cells, prostate cancers also metastasize primarily to skeletal regions suggesting that the elevated extracellular Ca^2+^ environment of the bone may provide a favorable niche for its localization and progression. In two highly metastatic prostate cell lines (PC-3 and C4-2B cells), elevated CaSR expression and increased proliferation was demonstrated compared to a non-skeletal metastatic, epithelial-derived prostate cell line (Liao et al., 2006). CaSR knockdown resulted in reduced PC-3 cell proliferation *in vitro* and reduced metastatic progression *in vivo*. The positive effect of CaSR activation on PC-3 cell proliferation was due to increased inhibition of cyclic AMP accumulation and elevated expression of cyclin D1, a protein required for cell cycle transition. Additionally, the effects of CaSR activation of PC-3 cell attachment and migration were attributed to activation of the Akt signaling pathway (Liao et al., 2006). Moreover, in human prostate cancer tissue sections and microarrays, CaSR expression was reported in both normal prostate and in primary prostate cancer samples as assessed by immunohistochemistry—but metastatic prostate cancer tissue obtained from bone had higher CaSR expression than primary prostate cancer tissue (Feng et al., 2014). Importantly, CaSR expression in primary prostate cancers of patients with metastases to tissues other than bone was not different from that in primary prostate cancer of patients with or without bony metastases. CaSR expression in cancer tissue was not associated with the stage or status of differentiation of the cancer (Feng et al., 2014). Taken together, these results suggest that the CaSR may have a role in promoting metastasis of prostate cancer to bone.

A link has also been indicated for the CaSR in renal carcinoma metastasis. To assess the role of the CaSR in kidney cancer, (Joeckel et al., 2014) distributed 33 matched specimens of normal and tumor tissue and 9 primary cells derived from renal cell carcinoma patients into three categories: non-metastasized, metastasized into the lung, and metastasized into bones during a 5-year period after nephrectomy (Joeckel et al., 2014). Thirty percent of renal cell carcinomas resulted in bone metastasis. Transcript and protein expression analysis showed the highest CaSR expression in samples obtained from patients with bone metastases. Ca^2+^-induced enhancement of renal cell carcinoma cell migration and proliferation were only identified in cells obtained from patients with bone metastases and absent in the other two groups. Analysis of intracellular signaling mechanisms of metastasizing carcinoma cells revealed that CaSR activation resulted in enhanced activity of Akt, PLCγ-1, p38α, and JNK signaling pathways, whereas PTEN expression was abolished. Based on these intriguing findings, it was suggested that CaSR expression levels could be utilized as a novel prognostic marker for predicting renal carcinoma cell bone metastasis (Joeckel et al., 2014).

## The CaSR and cell adhesion proteins—the integrins

### Integrin expression, activation, and bi-directional signaling

Our previous work delineated a novel interaction between the CaSR and a class of cellular adhesion proteins known as the integrins (Tharmalingam et al., 2011). Identification of the integrins as CaSR interacting proteins was initially determined in rat thyroid carcinoma cells from CaSR immuno-affinity pull-down experiments combined with mass spectrometry. Pharmacological stimulation of the CaSR using the positive allosteric modulator NPS R-568 potently enhanced thyroid carcinoma cell adhesion and migration. Evidence that these effects occurred via the integrins was indicated by the demonstration that the enhancement of cell-ECM adhesion by NPS R-568 could be largely blocked by the addition an integrin blocking peptide (GRGDSP) that competes with fibronectin for β1-integrin binding. Additional confirmation was obtained whereby shRNA-mediated knock down of β1-containing integrins reduced CaSR-mediated cell adhesion and migration. We concluded that (a) the CaSR is associated with β1-containing integrins in a macromolecular protein complex and (b) that stimulation of the CaSR promotes cell adhesion and migration via integrin activation (Tharmalingam et al., 2011).

The integrins are a family of proteins that mediate cell-to-cell contact by binding to the ECM. Integrins are cell adhesion molecules that interact with the ECM to provide a dynamic link between the extracellular adhesion molecules and the intracellular actin cytoskeleton. These proteins are unique in that they are able to bind to ECM proteins and induce intracellular signaling cascades, and are also capable of conveying intracellular signaling messages to the ECM by modulating their binding affinity for the ECM. By mediating cell-ECM interactions integrins play an essential role in cellular migration. They are also involved in other cellular processes including cellular differentiation, proliferation, embryogenesis, CNS development, inflammation and cancer (Iwamoto and Calderwood, 2015; Maartens and Brown, 2015; Wang et al., 2015).

**Figure 1:**
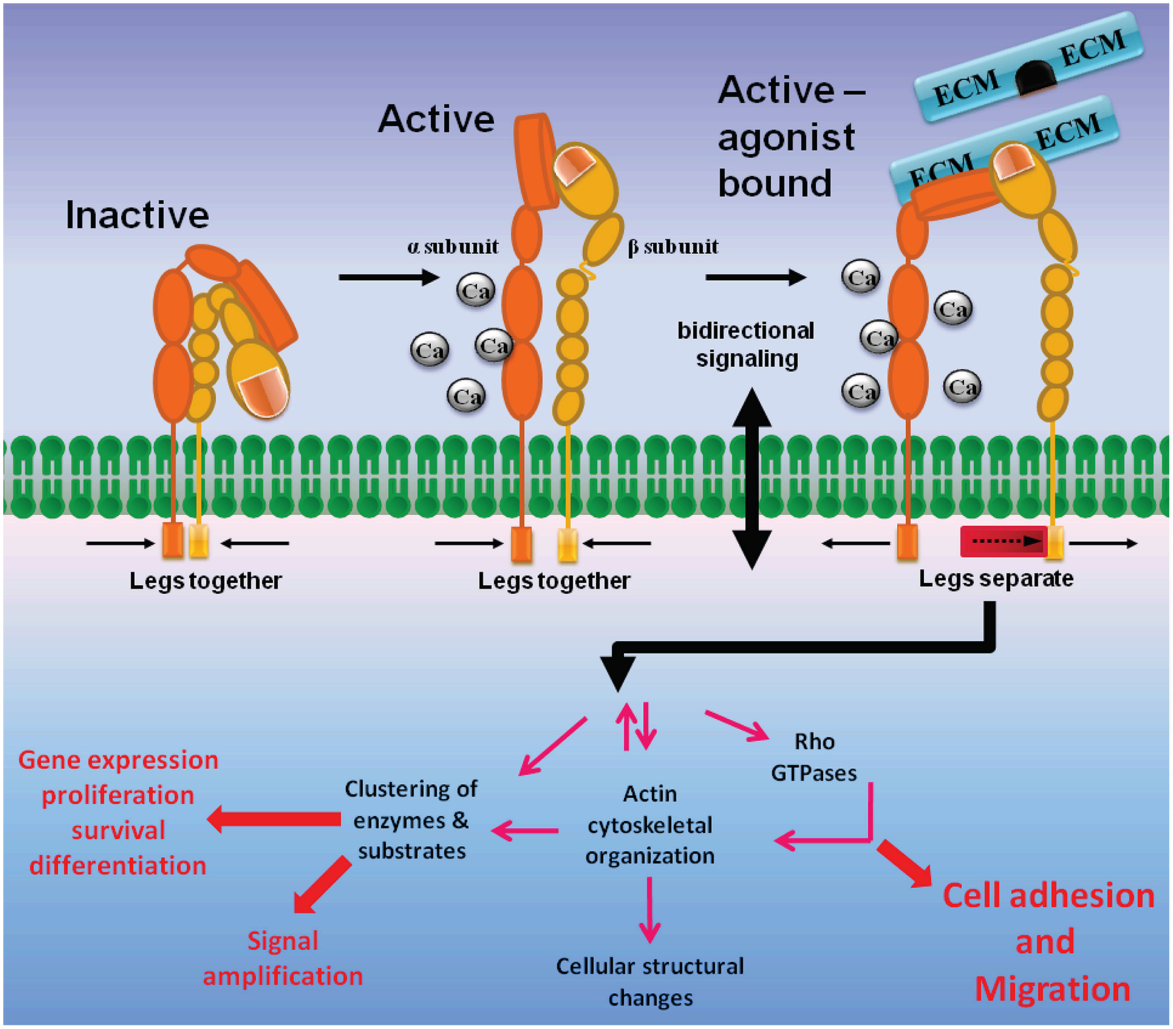
**Integrin structure and bidirectional signaling**. Integrins are membrane-spanning glycoprotein heterodimers that mediate cell adhesion and migration. The large extracellular domains contain sites for ligand and cation binding, while the small intracellular region senses changes in intracellular signaling pathways that convey information to the ECM via conformational modifications. Inactive integrins are present in a bent conformation (low affinity, left). Intracellular activation signals induce integrins to attain an upright position with a dual extended head-piece arrangement (middle). The upright conformation is characterized by an intermediate affinity state and a high affinity, ligand-bound state where the integrin α and β subunit tails are separated. Integrin inside-out signaling is achieved when intracellular signaling proteins push the β integrin leg away from the α integrin subunit, allowing for formation of an integrin high affinity activation state (right). Stabilization of integrin binding to ECM initiates outside-in signaling, where the integrins mediate downstream signaling that includes focal adhesion and actin stress fiber formations, activation of Rho GTPases, and cellular structural changes, etc., which ultimately leads to promotion of cell adhesion and migration.

Integrins are heterodimers of type 1 membrane-spanning glycoproteins composed of an α and a β subunit (Figure 1). In mammals, there are 18 α and 8 β subunits that form 24 heterodimeric pairs (see Campbell and Humphries, 2011 and Maartens and Brown, 2015 for reviews). Both α and β integrin subunits are characterized by a very large extracellular domain of 700–1100 residues, a single transmembrane domain, and a short cytoplasmic domain of 30–50 residues, with both subunits held together via non-covalent interactions. The outermost N-terminal region of the α integrin subunit that faces the extracellular side of the cell is composed of a head structure with seven-bladed β-propellers, which connects to the leg structure composed of a thigh, calf-1 and calf-2 domains (Barczyk et al., 2010). A region between the thigh and calf-1 domain known as the “α subunit genu” acts as a pivot point for integrin domain extension.

In the resting state integrins adopt a conformation where the integrin leg structures are severely bent, generating a closed topology where the head domain are juxtaposed to the leg regions close to the cell membrane (Figure 1). In this conformation the integrin ligand binding sites are not optimal for binding ECM proteins, and therefore the bent conformation represents a low-affinity state. The presence of ECM ligands induces an intermediate-affinity conformation in which a switch-blade like extension of the head region shifts away from the legs (Luo and Springer, 2006; Barczyk et al., 2010). Destabilization of the α/β integrin association fully activates the integrins into a high-affinity conformation (O'Toole et al., 1991; Lu et al., 2001), and transmembrane domain separation is necessary for integrin signaling (Wang and Luo, 2010).

From the intracellular side, integrins do not bind actin directly, but rather indirectly by proteins they recruit with their cytoplasmic tails. The dynamic complex of proteins at cell–ECM adhesions has been termed the integrin adhesome. A crucial adhesome component is the cytoskeletal-associated protein talin, which can simultaneously bind the cytoplasmic tail of integrin β subunits and actin, as well as coordinate the solicitation of other proteins to the adhesome (Critchley, 2009). Integrin inside-out signaling is mediated by the binding of talin to the β integrin C-terminal tail, a process that is controlled by the small GTPase Rap1 and its effectors such as RapL and RIAM (Mehrbod et al., 2013). Rap1 is activated by various GPCRs that are activated by chemoattractant stimulation. Stimulation of membrane spanning receptors mediates cell adhesion and migration by activating signaling cascades that lead to integrin inside-out signaling (Mehrbod et al., 2013). Upon separation of the integrin α and β subunits, the integrins adopt a high affinity active conformation that allows binding of ECM proteins. Ligand (ECM) occupancy and integrin clustering in-turn activates intracellular signaling cascades that mediate cell cytoskeletal changes, a process known as integrin outside-in signaling (Figure 1).

Upon binding to ECM proteins, integrin dimers cluster together laterally on the membrane via cell surface insertion of integrins localized in caveolae-rich microdomains (Maheshwari et al., 2000). Integrin clustering induces actin filament assembly and reorganization of actin filaments into larger stress fibers in turn causes further integrin clustering, providing a positive feedback loop that maintains enhanced matrix binding. The aggregation of ECM proteins, integrins, cytoskeletal proteins, and signaling kinases forms unique structures known as focal adhesion complexes (Parsons et al., 2010). In particular, integrin ligation and clustering activates focal adhesion kinase (FAK), which in-turn activates Src-family kinases (Mitra and Schlaepfer, 2006; Hu and Luo, 2013). Activated Src phosphorylates cytoskeletal proteins such as paxillin, actin bundling protein α-actinin and vinculin to form nascent adhesion structures. Binding of these initial cytoskeletal proteins recruits further actin nucleating proteins that result in focal adhesion complex and actin stress-fiber formation. These cytoskeletal changes mediated by integrin outside-in signaling ultimately allow cells to adhere and spread over the ECM proteins.

Intriguingly, and also similar to the CaSR, the integrins contain multiple divalent cation binding sites in the extracellular domain (Xiong et al., 2002). The integrin cation binding sites can be occupied by Ca^2+^ or by Mn^2+^ ions. However, the integrins differ from the CaSR in that the cations are necessary but not sufficient for integrins to convert from the inactive bent conformation into the active extended conformation. Both the presence of cations bound to the multiple cation binding sites is required, along with the direct physical association with ECM ligands for integrins to attain the extended structure and concomitant activation (Dai et al., 2015). Based on these features, *we hypothesize that a rise in extracellular Ca*^2+^ *ions would serve to both prime the integrin heterodimer and activate the CaSR (see further discussion below)*.

Integrins are crucial players in several aspects of bone tissue biology (see Marie et al., 2014 for review). Several integrin heterodimers including α5β1 integrin, α2β1 integrin, and α4β1 integrin have been shown to play important roles in bone formation and maintenance, and in bone cell differentiation (Hamidouche et al., 2009; Guan et al., 2012; Marie, 2012). In general, activation of integrins on osteoblasts leads to cell differentiation. In bone (and other tissues) ECM–integrin binding induces the recruitment and phosphorylation of focal adhesion kinase and activation of phosphatidylinositol 3-kinase (PI3K) and mitogen-activated protein kinase (MAPK) ERK1/2, leading to activation of the transcription factor RUNX2 and osteoblast differentiation.

Of special interest here in light of the identification of the CaSR- β1 integrin complexes, is that β1-containing integrins play an important role in osteoblast differentiation. Direct evidence supporting this concept comes from mice expressing a dominant-negative β1 integrin subunit in mature osteoblasts. These mutant mice show reduced bone mass, defective bone formation and loss of ICAP-1, an important modulator of β1 integrin activation resulting in defective osteoblast differentiation and delayed bone formation (Globus et al., 2005; Brunner et al., 2011). The a5β1 integrin heterodimer which binds to the ECM protein fibronectin, is essential for osteoblast formation, and α5 integrin suppression reduces osteoblast differentiation, whereas its overexpression facilitates osteoblast differentiation in cultured mesenchymal skeletal cells. These effects are mediated via FAK/MAPK ERK1/2 signaling (Hamidouche et al., 2009). It has also been shown that integrin αvβ3 is critical for CYR61-mediated BMP-2 expression and subsequent osteoblastic differentiation through activation of the ILK/MAPK ERK1/2 signaling pathway in osteoblasts (Su et al., 2010). Similarly, α2β1, a major receptor for collagen type 1, was reported to control collagen synthesis in osteoblasts in mice (Stange et al., 2013). This integrin heterodimer plays a role in mesenchymal skeletal cells osteogenic differentiation and survival through activation of ROCK, FAK, and MAPK ERK1/2 signaling. Together, these findings provide insight into the role of integrin-mediated signaling pathways in the control of osteoblast differentiation, fate, and function (Marie et al., 2014).

### Integrins and metastasis

Integrins have been intensively studied in the context of cancer with hundreds of literature citations on the topic; therefore, we will restrict our discussion here to the most pertinent findings on the association of β1-containing integrins and metastasis to bone.

β1 integrins are activated in metastatic prostate cancer cells and mediate increased prostate cancer cell metastasis to lymph nodes and bone. Dissemination of prostate cancer cells to the bone marrow occurs early in the disease. Cancer cell proliferation and active metastases after a period of undetectable disease is known as tumor dormancy. Dormancy-reactivation has been studied in patient-derived xenograft lines where the propensity to proliferate through tumor cell contact with each other, and with bone marrow stroma was demonstrated (Ruppender et al., 2015). Proliferating prostate cancer cells displayed tumor cell-cell contact and integrin clustering after ECM contact (e.g., fibronectin) in bone marrow stroma. Activation of myosin light chain kinase, a downstream effector of integrin-β1 and TGF- β2, in non-proliferating cells promoted cell proliferation. This activity leads to the deactivation of growth suppressor like E2F4 and activation of cell cycle regulator CDK6 thereby promoting cell proliferation (Ruppender et al., 2015). These data illustrate the involvement of integrins in the escape of prostate cancer cells from dormancy.

Similar to CaSR expression in metastatic prostate cancers, β1-containing integrins have been shown to be constitutively activated in prostate cancer cell lines with high metastatic potential, but not in cancer cell lines with low metastatic potential (Lee et al., 2013). Activation of β1 integrins promotes resistance to anoikis. Anoikis is a form of programmed cell death that is induced by anchorage-dependent cells detaching from the surrounding ECM. Usually cells stay close to the tissue to which they belong since the communication between proximal cells as well as between cells and ECM provide essential signals for growth or survival. When cells are detached from the ECM, there is a loss of normal cell–matrix interactions, and they may undergo anoikis. However, metastatic tumor cells may escape from anoikis and invade other organs. Resistance to anoikis occurs via stimulation of FAK/Akt-mediated survival pathway and neutralizing β1 integrin activation using an anti-β1 integrin antibody reduced prostate cancer metastasis *in vivo* (Lee et al., 2013). In addition, talins which are adaptor proteins that regulate focal adhesion signaling by conjugating integrins to the cytoskeleton, bind integrins and are essential for integrin activation. In this context, talin has been shown to be important for β1 integrin activation, and Cdk5-mediated phosphorylation of talin leading to β1 integrin activation was shown to increase the metastatic potential of prostate cancer cells (Jin et al., 2015).

Integrins are also involved in breast cancer metastasis. Bone metastasis affects more than 70% of advanced breast cancer patients, but the underlying molecular mechanisms remain unclear. Although some evidence suggests that integrins mediate adhesion of malignant cells to bone ECM, their role during bone colonization is also not fully understood. The role of β1 integrins in bone colonization has been investigated *in vitro* and *in vivo* in tissue-engineered humanized bone models (Thibaudeau et al., 2015). *In vitro*, bone- metastatic breast cancer cells with suppressed integrin β1 expression showed reduced attachment, spreading, and migration within human bone matrix compared to control cells. Cell proliferation *in vitro* was not affected by β1 integrin knockdown, but tumor growth *in vivo* within humanized bone microenvironments was significantly inhibited upon β1 integrin suppression. Tumor cells invaded bone marrow spaces in the humanized bone and formed osteolytic lesions whereas osteoclastic bone resorption was, however, not reduced by β1 integrin knockdown (Thibaudeau et al., 2015). These observations indicate that β1-containing integrins likely play a prominent role in bone colonization.

Some data are also available linking integrins and bone metastasis in renal carcinomas. Renal carcinoma cells that form bone metastasis constitutively activate osteoclasts, which then induce bone resorption and release pro-proliferative factors. As noted above, this phenomenon also occurs in bone metastasis of other types of tumors including breast and prostate cancer. Constitutive activation of osteoclasts leads to the release of fibronectin and collagen which can facilitate cancer cell attachment via integrins to ECM and vascular endothelial cells. Cell adhesion of bone metastasizing cells to fibronectin and to collagen I (but not adhesion to collagen IV) is high and bone has a high concentration of these two ECM proteins (Ode et al., 2010). In one study, 9 renal cell carcinoma primary cell lines and 30 renal cell carcinoma tissue specimens (normal and tumor tissue) collected from 3 patients with no metastasis, and 10 samples with lung or bone metastasis within 5 years after nephrectomy were collected and analyzed (Haber et al., 2015). Compared to renal cell carcinoma cells from patients without metastasis, the migration of cells from patients with bone metastasis was enhanced 14-fold, and adhesion to fibronectin and collagen I were both enhanced 6-fold. Notably, proliferation was decreased in metastasizing cells. In addition, elevated activity of Akt and FAK and integrin α5 expression, and reduced PTEN expression was observed in primary cells from patients with bone metastasis compared to non-metastasizing cells. Thus, (Haber et al., 2015) concluded that increased expression of integrin α5 and downstream signaling via Akt could help tumor cells to adhere to ECM proteins and facilitate migration to bone tissue.

**Figure 2:**
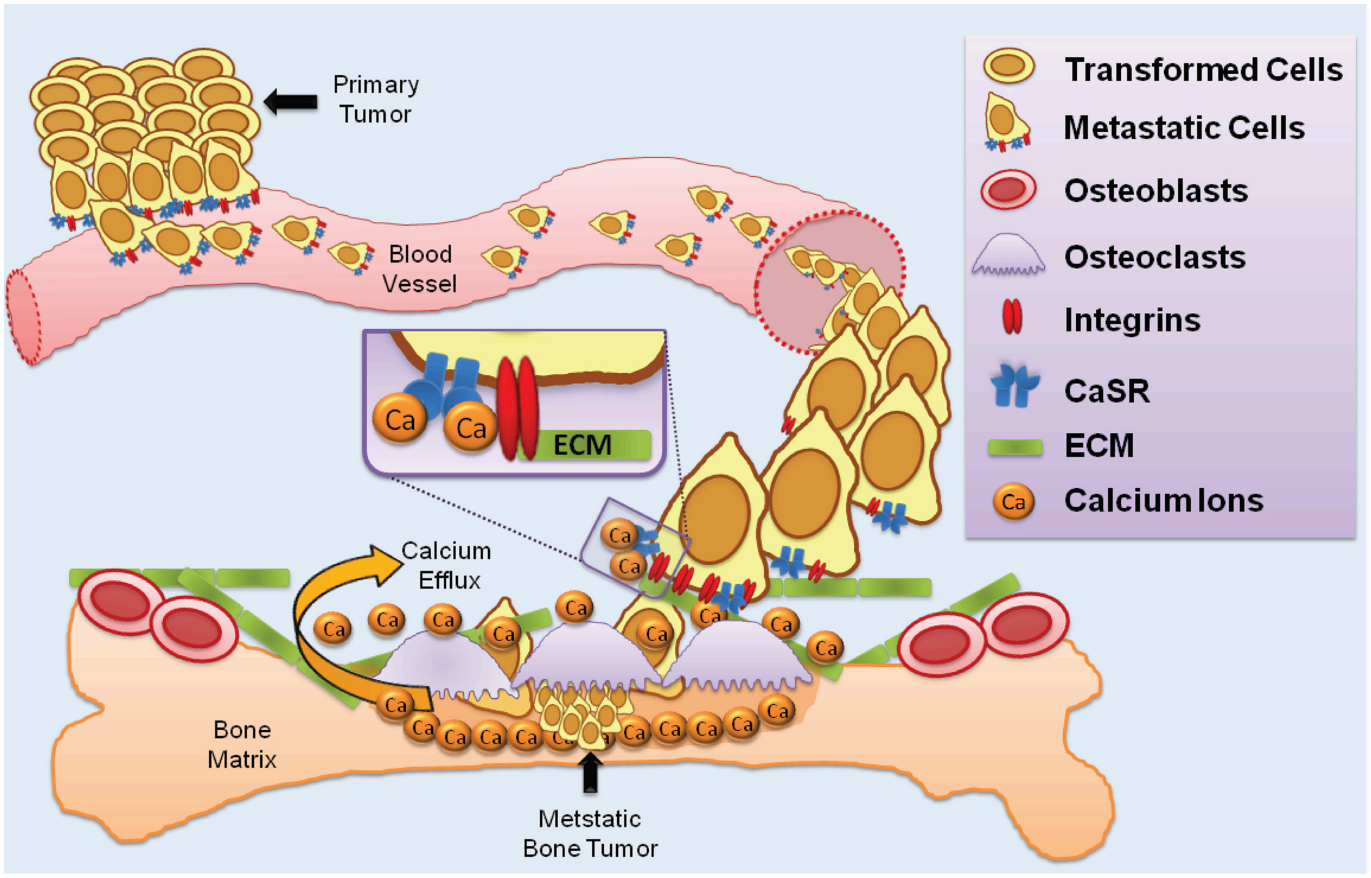
**CaSR and integrins in cancer metastasis to bone**. The diagram illustrates a proposed multi-step process whereby primary tumor cells enter the vasculature and colonize resorbing regions in bone. Tumor cells with elevated CaSR and integrin expression intravastates blood vessels and circulate in the bloodstream. Resorbing bone houses osteoclasts which release Ca^2+^; elevated extracellular Ca^2+^ is detected by the CaSR which activates integrin heterodimers to promote binding to ECM proteins such as collagen and laminin.

In summary, three lines of evidence lend support to the hypothesis that CaSR-integrin complexes may be a factor in some forms of cancer metastasis: (1) the findings outlined above and others indicating a role for β1-containing integrins in cancer metastasis to bone, (2) the considerable body of evidence demonstrating elevated CaSR expression in prostate, breast, and kidney cancers that metastasize to bone, and (3) the presence of CaSR-β1 integrin macromolecular complexes in thyroid carcinoma cells (Tharmalingam et al., 2011). We postulate that in circulating tumor cells, CaSR-integrin protein complexes could function together as a cell surface detection mechanism for attaching to the ECM components of the bone microenvironment (Figure 2). The CaSR could act as the calcium sensor and become activated by the (presumed) high extracellular Ca^2+^; in bone tissue this would be followed in turn by activation of the associated integrins on the tumor cell to signal attachment to bone ECM proteins such as fibronectin and collagen. It should be emphasized that this proposed sequence of events is speculative and further work is needed to rigorously test the idea.

## The CaSR and integrins in the developing CNS

### Developmental expression of the CaSR and integrins in the CNS

CaSR expression in the central nervous system (CNS) was first demonstrated using a rat striatal cDNA library where it was determined that the receptor is expressed in both neurons and glia (Ruat et al., 1995). In adults, the CaSR is localized to nerve terminals with discrete punctate expression. Upon depolarization, the synaptic space contains elevated Ca^2+^. Due it is localization within nerve terminals, the CaSR may regulate neurotransmitter disposition in response to synaptic Ca^2+^ changes (Ruat et al., 1995; Ruat and Traiffort, 2013). In the immature brain the receptor is expressed in several regions including the orbital cortex, cerebellum (see below), and hippocampus (Ferry et al., 2000; Tharmalingam et al., 2016). Analysis of rat hippocampal mRNA and protein expression showed that peak CaSR expression occurs from postnatal day 12 to day 24, followed by a 3-fold decrease to adult levels (Chattopadhyay et al., 1997). Cellular analysis demonstrated CaSR expression in pyramidal cells of all the layers of hippocampus and in granule cells of the dentate gyrus.

Studies on of CaSR knockout mice during postnatal brain development have indicated that the CaSR plays an important role in the differentiation of numerous brain cell types. For example, CaSR null mice exhibited developmental delay due to impaired differentiation as assessed by reduced expression of proliferating cell nuclear antigen, neuronal specific nuclear protein (neuronal differentiation marker), glial fibrillary acidic protein (glial differentiation marker), and myelin basic protein (oligodendrocyte differentiation marker) when compared to age-matched wild-type littermates (Liu et al., 2013). Brain weight and size were drastically reduced in the CaSR null mice and cell proliferation was also decreased in the CaSR knockout animals. Concurrent deletion of the CaSR with parathyroid hormone gene corrected for the effects of hyperparathyroidism, hypercalcemia, hypophosphatemia, and whole-body growth retardation (Liu et al., 2013). However, further analysis of these animals established that brain cell proliferation was normal whereas differentiation was greatly reduced in the CaSR-PTH double knockout animals. The role of the CaSR in differentiation vs. proliferation was also investigated using cultured neural stem cells derived from the subventricular zones of CaSR null neonatal mice, which exhibited normal proliferation capacity but decreased differentiation capacity when compared with wild-type animals (Liu et al., 2013). Taken together, the CaSR is crucial for differentiation of neurons, glia and oligodendrocytes. Delayed brain development in CaSR null newborn mice is therefore the result of direct effects of the CaSR on cell differentiation, and indirect CaSR effects on cell proliferation via dysregulation of parathyroid hormone secretion and serum Ca^2+^ (Liu et al., 2013).

The CaSR expression pattern in adult rodent brain differs substantially from the immature brain. The regions of moderate to high expression during development do not express the receptor, or express it at very low levels, in the adult rodent brain. However, the CaSR is expressed in a few regions of the adult CNS including the olfactory bulbs, area postrema, and the subfornical organ (Ferry et al., 2000; Yano et al., 2004). The subfornical organ is a hypothalamic thirst center; its neurons project onto supraoptic and paraventricular nuclei where they control the activity of vasopressin-secreting neurons (Ruat and Traiffort, 2013). Expression in the subfornical organ suggests that the receptor is also involved in central fluid and electrolyte balance (Yano et al., 2004). The adult cerebral arteries also display an intense network of CaSR immunoreactive fibers demonstrating possible functional role in vessel innervation. Studies on adult CaSR neuronal expression have determined that the CaSR controls neuronal excitability by regulating ion channel function (Phillips et al., 2008; Vyleta and Smith, 2011). These findings reinforce the idea that the CaSR modulates Ca^2+^-dependent neuronal functions in both the immature and in the adult brain.

β1 integrins are expressed in all cell types in the CNS and integrin heterodimers containing the β1 integrin are indispensable for many functions in the brain where the β1 subunit forms obligate heterodimers with numerous α integrin subunits to create functional receptor complexes. Each brain region and cell type expresses its own repertoire of α integrin subunits resulting in different integrin receptor expression profile and function (reviewed by Pinkstaff et al., 1999). In the developing nervous system, spatial and temporal changes in integrin expression parallel changes in neurogenesis, differentiation, and migration (Schmid and Anton, 2003). In early CNS development, neuronal migration during establishment of the cerebral and cerebellar cortex requires β1 integrin-mediated adhesion and migration (Hatten, 1999). Neurons originating from the ventricular zone migrate radially using glial cells and detach from the glial fibers at the cortical plate to establish connections at their final resting position. Loss of β1 integrins in neurons results in grossly disorganized structures in the most superficial layer of the cortex (Milner and Campbell, 2002), and selective deletion of β1 integrins in glia resulted in failure of glial cell contact formation with the meningeal basement membrane and blocked neuronal radial migration (Blaess et al., 2004; Frick et al., 2012). Therefore, normal assembly of the cerebral and cerebellar cortex requires β1 integrin mediated neuronal migration and glial adhesion (Milner and Campbell, 2002).

### CaSR-integrin complexes in cerebellar development

**Figure 3:**
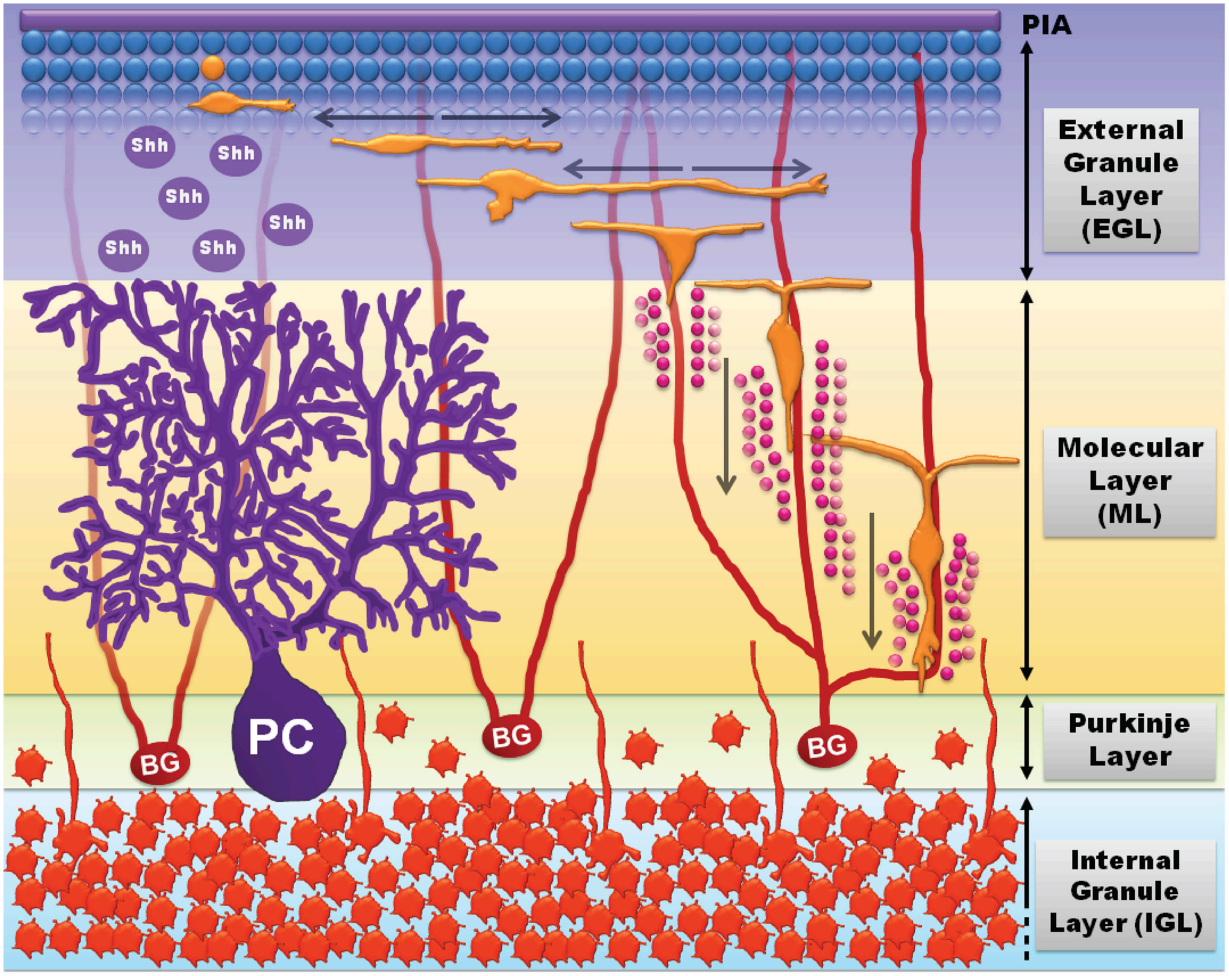
**Cerebellar development and GCP migration (adapted from Tharmalingam, 2014)**. GCPs (blue spheres, top) proliferate in response to growth signals including sonic hedgehog (shh) released by Purkinje cells (PC, purple). Mitotic GCPs exit the cell cycle to become migratory post-mitotic bipolar GCPs (orange) that undergo tangential migration in the external granule-cell layer (EGL). The bipolar migratory GCPs sprout a third process and attach to Bergmann glia (BG) fibers (red) to undergo radial migration through the molecular layer (ML) and the Purkinje layer (PL), and into the internal granule-cell layer (IGL; mature granule neurons, dark orange). GCPs undergo radial migration by sensing extracellular matrix cues (laminin, vitronectin, etc.) and radially expanding transglial Ca^2+^ waves (pink circles) released by Bergmann glial processes.

The well-established migrational patterns of immature cerebellar granule-cell precursor neurons (GCPs) in the early postnatal cerebellum provides an excellent model system to delineate the molecular properties of neuronal migration. GCPs originate in the rhombic lip during late embryonic stages and then migrate rostrally to a secondary germinal layer, known as the external granule-cell layer of the cerebellum, where they continue to proliferate after birth. In rodents GCPs proliferate in the periphery of the external granule layer-cerebellar basement membrane interface over postnatal days 5–20 (Figure 3). Various factors such as laminin (expressed on the basement membrane), sonic hedge-hog (released from Purkinje cells), brain-derived neurotropic factor (released from Bergmann glia), and insulin-like growth factors contribute to GCP proliferation. GCPs that have proliferated and exited the cell-cycle undergo tangential migration within the external granule layer (Figure 3). Tangentially migrating GCPs attach to the Bergmann glial processes and undergo radial migration whereby the GCPs migrate radially along the Bergmann glia fibers through the cerebellar molecular layer and the Purkinje cell layer, and into their final resting position in the internal granule-cell layer (Chedotal, 2010). By 3 weeks after birth GCP proliferation ceases and the remaining GCPs complete migration into the internal granule layer, which is retained in the mature brain while the external granule layer disappears. In the adult CNS cerebellar granule cells are the most numerous class of neurons in the vertebrate brain.

The role of β1 integrins in cerebellar GCP proliferation and migration has been well documented. Potent stimulants of GCP proliferation such as sonic hedgehog and SDF-1 have been shown to induce GCP proliferation only in the presence of β1 integrin mediated adhesion to laminin (Klein et al., 2001; Blaess et al., 2004). β1 integrins control GCP migration by binding to ECM proteins such as laminin, vitronectin, reelin, etc., which aid in the homing of GCPs from the external granule layer to the internal granule layer (Pons et al., 2001; Borghesani et al., 2002; Porcionatto, 2006). Thus, GCP migration relies heavily on integrin activation. Notably, defects in GCP migration result in medulloblastoma, the most common cause of malignant brain tumors in young children (see below).

We have recently demonstrated that the CaSR, together with integrins, functions as a cell-surface chemoattractant receptor expressed on the surface of cerebellar GCPs to facilitate GCP differentiation into mature granule cells, and to simultaneously guide their movement during cerebellar maturation (Tharmalingam et al., 2016). A robust but transient increase in CaSR expression in GPCs was observed that peaked during the second postnatal week, the period of maximal GPC migration. Our results support a model whereby the CaSR acts as a chemoattractant sensor to detect a gradient of extracellular Ca^2+^, while integrin heterodimers in conjunction with ECM proteins function as the driving force to mediate cell movement (Tharmalingam et al., 2016). The data also indicated that CaSR-mediated stimulation of ERK2 and Akt phosphorylation provides a biochemical link between Ca^2+^-sensing and the integrin-mediated movement of GCPs (**Figure 5**; also see further discussion below).

A key question to be addressed is, what is the source of the CaSR stimulus in the immature cerebellum? One possibility is that activation of the CaSR may be mediated by local extracellular Ca^2+^ released by the Bergmann glia. Tangentially migrating unipolar GCPs in the external granule layer sprout a third axon that allows the GCPs to attach to Bergmann glial fiber tracts to undergo radial migration into the internal granule layer. Bergmann glial fibers have been shown to generate radially expanding transglial Ca^2+^ waves that initiate at a central point and expand radially to encompass multiple Bergmann glial processes (Hoogland et al., 2009). The Ca^2+^ waves generated by Bergmann glia could locally activate the CaSR expressed on GCPs to promote radial migration. Another potential source of CaSR activation may be from polyamines. Polyamines such as spermine and spermidine are potent agonists of the CaSR and polyamine concentrations are elevated during postnatal cerebellar development and then subsequently decline to adult levels (Jasper et al., 1982). The period of increased polyamines corresponds to the peak period of CaSR expression and GCP migration. Moreover, the initial step in the synthesis of the polyamines is mediated by the enzyme ornithine decarboxylase. In the cerebellum of rats injected with α-difluoromethylornithine, an inhibitor of ornithine decarboxylase, granule cells fail to migrate properly and the development of the cerebellar cortex is stalled in an immature state (Bartolome et al., 1985). Taken together these findings indicate that polyamines might act alone, or in conjunction with Ca^2+^ ions, to stimulate the CaSR and guide GCP migration in the developing brain.

### Cerebellar granule cells and medullablastoma

Defects in GCP migration result in medulloblastoma, the most common cause of malignant brain tumors in children under four (Hatten and Roussel, 2011; Remke et al., 2013; Macdonald et al., 2014). Medulloblastoma is a highly heterogeneous group of tumors characterized by aberrant GCP proliferation and migration. Unlike most neuronal populations, GCPs remain mitotically active after birth (Roussel and Hatten, 2011). Impaired migration causes GCPs to remain longer in the proliferative-niche of the external granule layer where they are more prone to transformational insults due to increased exposure to proliferation-inducing molecules. There are currently no effective treatments for medulloblastoma. Although a role for the CaSR in medulloblastoma has not been established, the identification of key proteins regulating GCP migration and progression is a major goal in medulloblastoma research, and could lead to novel approaches for the development of effective therapeutics.

Medulloblastoma has been modeled *in vitro* using DAOY cells which are transformed human cerebellar granule cells. Wei et al. (2015) studied the function of the CaSR in these cells and compared them to normal cerebellar granule cells in culture (Wei et al., 2015). They examined the CaSR in conjunction with OGR1 (also known as GPR68), an extracellular proton-sensing GPCR coupled to Gq and the release intracellular Ca^2+^. In addition to protons, this receptor is also activated by the benzodiazepine anxiolytic drug lorazepam (Huang et al., 2015). Previous work had established that OGR1 activation in DAOY cells leads to complex intracellular Ca^2+^ signals and activation of the ERK signaling pathway, thereby providing a mechanistic explanation of how the acidic environment may influence transformed cell function and/or survival. This action is lost on differentiation, suggesting a link between OGR1 activity and proliferative behavior of the transformed neurons (Huang et al., 2009). Curiously, Ca^2+^ signaling through OGR1 and CaSR appears to be opposite in the two cell types. In normal granule cells, OGR1-dependent Ca^2+^ signaling is slow and small while CaSR-dependent intracellular Ca^2+^ signaling is fast and large, whereas the opposite occurs in DAOY cells (Wei et al., 2015). It was suggested that the lack of inhibition of OGR1 signaling by CaSR in DAOY cells is likely due, at least in part, to low expression of functional CaSR in these cells, given that knockdown of CaSR in granule cells also leads to disinhibition of OGR1 signaling. Moreover, CaSR-dependent inhibition of OGR1 activity is absent in DAOY cells and intracellular acidification, which may accompany extracellular acidosis, inhibited CaSR responses but potentiated OGR1 responses (Wei et al., 2015).

OGR1 is functionally expressed in brain tumor cells and changes in its activity may be relevant to acidosis in tumors. Both OGR1 and CaSR have been implicated in several brain disorders that are exacerbated by changes in the concentrations of extracellular H^+^ and Ca^2+^ (Kingsley et al., 2007). The CaSR and OGR1 are also co-expressed in other tissues that experience extracellular acidification under physiological conditions including kidney, bone, and lung. Therefore, in addition to cerebellar neurons, a perturbation in the balance between OGR1 and CaSR signaling may contribute to the development and progression of other pathological conditions.

## Potential molecular mechanisms underlying CaSR-integrin interactions and cellular migration

**Figure 4:**
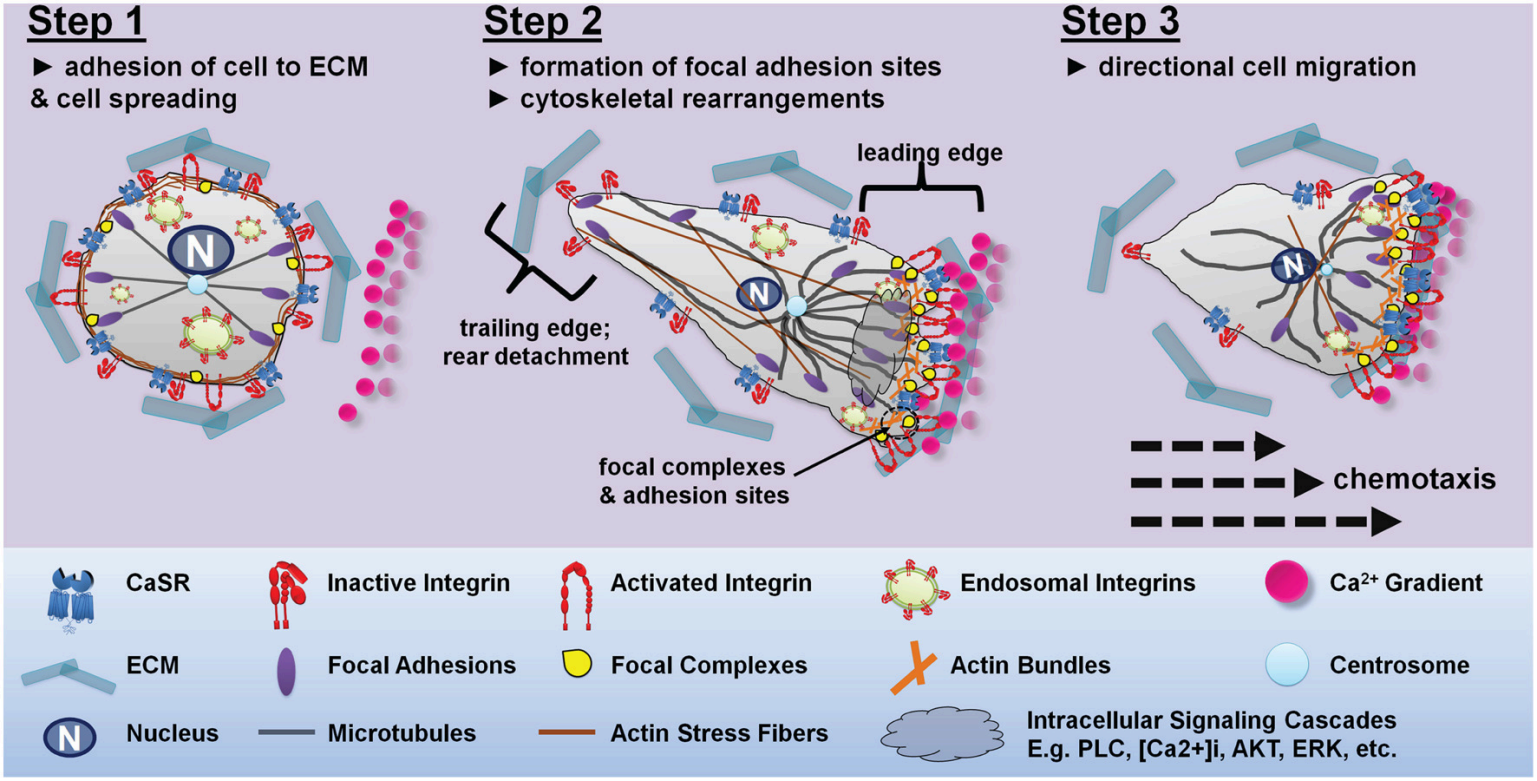
**Proposed mechanism for CaSR-integrin mediated directional cell migration toward a CaSR-agonist gradient**. Step 1: under resting conditions there is a homogenous distribution of integrins along the plasma membrane of the cell. Integrins present in the active confirmation are bound to the ECM proteins allowing the cell to spread evenly on the ECM substratum. In the absence of CaSR agonists, the integrins present in the CaSR-integrin complex adopt an inactive bent conformation. Step 2: exposure of the cell to a Ca^2+^ gradient results in CaSR-integrin mediated cell polarization. The CaSR-integrin complexes present proximal to the Ca^2+^ gradient uses the CaSR to sense the stimulus allowing for localized activation of intracellular signaling pathways. Activation of CaSR triggers PLC-IP_3_ mediated rise in intracellular Ca^2+^ causing integrin inside-out activation and elevated activated integrins at the site of CaSR stimulation. CaSR activation also promotes ERK2 and Akt-mediated trafficking and insertion of cytoplasmic endosomal integrins into the plasma membrane. Increased integrin-ECM interactions at the leading edge establishes cell polarity by allowing the formation of adhesion contacts and actin polymerization at the migrating front, while the tailing edge disassembles actin formation. Step 3: CaSR stimulation dependent redistribution of integrins and actin stress formation from the trailing edges to the migrating front is important for guided cell migration. The transfer of actin fibers to the leading edge of the cell, while limiting the level of integrins, actin and microtubules available in the retracting edge prompts directed cell migration toward the calcium gradient. The dynamic assembly and disassembly, together with actomyosin contractility moves the cell toward the Ca^2+^ gradient.

Cell migration is a multistep process that requires continuous coordinated formation and disassembly of cytoskeletal proteins (Webb et al., 2002). The migratory cycle progresses from activation of a chemoattractant receptor, protrusion extension toward a chemoattractant gradient, formation of stable adhesions at the leading edge of the protrusions via integrin engagement of ECM proteins, release of adhesions and retraction at the cell rear, and the translocation of cell body forward (Figure 4). Increased integrin-ECM ligation promotes outside-in integrin signaling characterized by focal adhesion complex formation and actin cytoskeletal polymerization (Vicente-Manzanares et al., 2009). These cytoskeletal changes drive the initial protrusion extension of the plasma membrane allowing the cell to spread. Cell polarity is achieved when the leading edge forms stable adhesion contacts via actin polymerization, while the trailing edge disassembles actin formation, and stable adhesion contacts serve as traction points for the propulsive forces that move the cell body forward (Webb et al., 2002). Release of integrin mediated adhesions at the rear allows translocation of the cell in the direction of the chemoattractant gradient (Figure 4).

The CaSR and integrins are both abundantly localized in caveolae-rich microdomains (Breitwieser, 2013; Iwamoto and Calderwood, 2015; Maartens and Brown, 2015). In cerebellar GCPs, this microdomain-type expression pattern is revealed in discrete puncta present throughout the cell cytosol. In GCPs, *activation of the CaSR resulted in increased cell migration by promoting cell surface expression of* β*1 integrins, but surprisingly, stimulation of the CaSR did not increase the cell surface expression of the CaSR itself* (Tharmalingam et al., 2016). This observation might be attributed to trafficking mechanisms which control receptor cell surface expression. CaSR and integrin cell surface expression is driven by agonist stimulation, in that activation of the surface receptor activates signaling cascades which allows further insertion of caveolae-rich microdomain containing receptors to the surface, thus allowing persistent signaling in the presence of chronic agonist stimulation (Grant et al., 2011; also see discussion below).

At the molecular level, one potential mechanism for functional cooperation between the CaSR and integrins is via a protein-protein interaction between the CaSR homodimer and the integrin heterodimers within a tetrameric complex, or as part of a larger complex containing additional proteins. A direct protein-protein interaction would require that the CaSR homodimer and integrin heterodimers are either physically in contact with each other, which has not yet been directly demonstrated, or present with additional proteins that influence each other by conformational changes within a macromolecular complex. In this scenario, we propose a working model whereby an agonist-induced conformational change in the CaSR induces flipping of the associated integrin heterodimers into an active conformation. Both the integrins and the CaSR possess large extracellular domains. Although no studies directly examining conformational changes in the CaSR have been reported, other members of this GPCR subfamily such as the GABA_*B*_ receptor and the metabotropic glutamate receptors, are known to undergo closure of their large extracellular Venus flytrap domains upon agonist binding (Tsuchiya et al., 2002; Pin et al., 2009). Activation of the CaSR could directly trigger integrin activation thereby inducing cellular adhesion, differentiation, or migration depending on the cellular environment.

**Figure 5:**
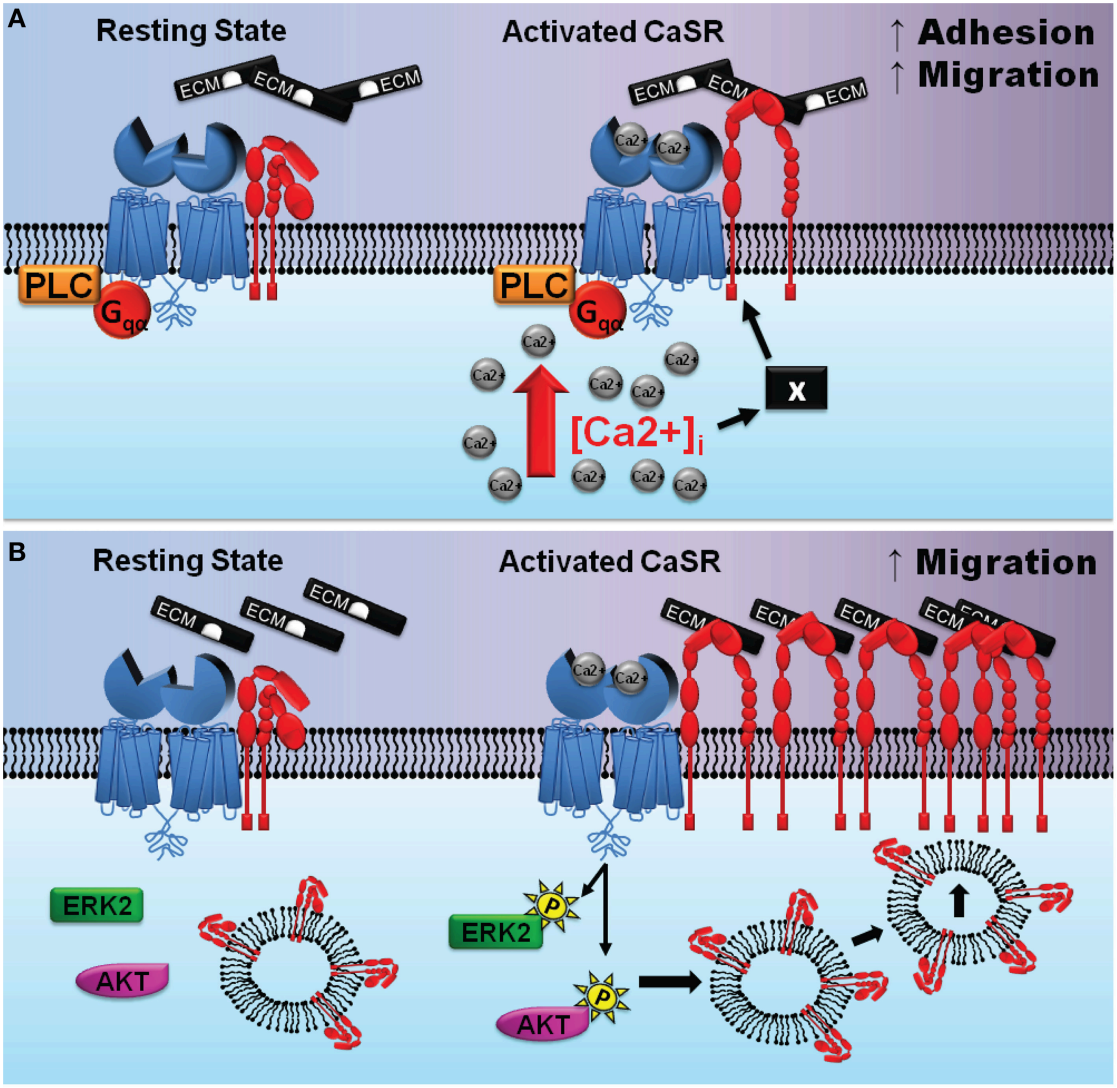
**Potential mechanisms of CaSR-integrin actions in cancer cells and the developing CNS. (A)** (adapted from Tharmalingam et al., 2011) depicts one possible scenario where the CaSR is associated with integrin heterodimers in a signaling complex in thyroid carcinoma cells. Left, the complex prior to CaSR stimulation; right, elevated extracellular calcium activate the CaSR causing its Venus flytrap domain to undergo a conformational change. This protein domain movement may induce integrins to flip it into an activated state thereby promoting binding to the ECM and enhanced cellular migration. The “X” denotes a hypothetical protein that may participate in facilitating the CaSR-mediated activation of integrins subsequent to increased [Ca ^2+^]_*i*_. **(B)** (adapted from Tharmalingam et al., 2016) depicts cross-talk between CaSR (blue), integrin heterodimers (red), and intracellular protein kinases in the developing cerebellum. Left, the CaSR/integrin complex prior to stimulation; right, elevated extracellular Ca^2+^ (and/or polyamines) activate the CaSR which then increases phosphorylation of ERK2 and Akt promoting β1 integrin cell surface expression. The accumulation of integrins at the leading edge of GCPs facilitates stable attachments between the GCPs and the ECM and enhances cellular migration.

An alternative mechanism to consider is a series of biochemical events that entail stimulation of CaSR/integrin complexes followed by the activation of intracellular signaling cascades. Activation of the CaSR is linked to several intracellular signaling pathways which could modify integrin conformation from within the cell. Our previous work in thyroid carcinoma cells provided evidence for an indirect mechanism in which stimulation of the CaSR induces activation of PLC and release of intracellular Ca^2+^, which in turn promotes cell adhesion and migration (Tharmalingam et al., 2011; see Figure 5A). Stimulation-dependent redistribution of integrins and actin stress formation from the trailing edges to the migrating front is important for chemoattractant-guided cell migration, and PLC activation and the release of intracellular Ca^2+^ have been shown to be important for integrin inside-out activation (Rowin et al., 1998). Specifically, a rise in intracellular Ca^2+^ increases cell adhesion and migration by specifying the location and timing of cell process retraction (Sjaastad et al., 1996; Valeyev et al., 2006); migrating cells display increased intracellular Ca^2+^ gradients which increase toward the rear of cells (Brundage et al., 1991; Brust-Mascher and Webb, 1998). Elevated intracellular Ca^2+^ has also been linked to increased contractility and adhesion disassembly mediated by calpain, a Ca^2+^ dependent protease (Doyle and Lee, 2002; Robles et al., 2003; Valeyev et al., 2006). Thus, a CaSR-induced rise in intracellular Ca^2+^ may promote integrin inside-out activation at the migrating front while simultaneously inducing adhesion disassembly at the rear of the cell, which then translates into cellular migration.

In contrast to thyroid carcinoma cells, CaSR-integrin actions mediating migration of GCPs during cerebellar development appears to be mediated not by phospholipase C, but rather by the ERK and Akt signaling (Tharmalingam et al., 2016). ERK2 and Akt signaling is known to promote plasma membrane insertion of β1 integrins localized in caveolae-rich endosomal microdomains (Maheshwari et al., 2000; Kinashi, 2005). Phosphorylation of ERK2 supports cell polarization by controlling the proper orientation of centrosomes, an important step for the accumulation of proteins involved in cell movement at the leading edge of the migrating cell (Bisel et al., 2008; Imamura et al., 2010).

We propose that stimulation of the CaSR activates phosphorylation of ERK2 and Akt which then signals the insertion of microdomains containing co-localized CaSR-integrin complexes (Figure 5B). Upon entering the plasma membrane, the CaSR agonists activate the newly inserted receptors, which then transactivates the integrins which causes engagement of ECM ligation. The ligated integrins may remain on the plasma membrane to mediate cell adhesion and migration, whereas the endocytotic mechanisms remove the CaSR from the cell surface and degrade the receptor via lysosomal enzymes. In this scenario, accumulation and clustering of integrins would take place at the cell surface, whereas the CaSR would serve as both the signal-sensing partner and as an integrin trafficking partner in the protein complex whose net cell surface expression may not change. In summary, in cerebellar neurons, CaSR-mediated activation of ERK and Akt may mediate trafficking of β1 integrins to the leading edge of the cell and facilitate the formation of a physical link between the ECM and focal adhesion complexes which act to promote neuronal migration.

## Conclusions, implications, and outstanding questions

1. The CaSR and GPRC6A are close structural paralogs that are widely expressed in many organs. Both receptors are activated and/or modulated by cations and amino acids.
2. The CaSR and integrins share no sequence homology but both are membrane proteins that function as dimers and possess multiple cation binding sites. Both proteins also undergo large conformational movements into an activated state after agonist stimulation. CaSR/integrin protein complexes have been identified in thyroid carcinoma cells and in cerebellar granule cells, and may exist in other tissues and cells.
3. The CaSR has been shown to play an essential role in cellular differentiation and migration in several cells and tissues, and CaSR-mediated cellular migration likely occurs, at least in some cells, via the activation of CaSR/integrin protein complexes. In thyroid carcinoma cells, the CaSR together with integrins facilitate cellular migration. In the developing cerebellum, activation of the CaSR induces neuronal differentiation and migration. Notably, stimulation of the CaSR in cerebellar neurons did not change the level of plasma membrane expression of the CaSR itself, but instead promoted the trafficking of integrins to the cell surface. Thus, it appears that cerebellar granule cell migration is mediated by a CaSR-induced increase of integrins to the neuronal plasma membrane.
4. In addition to thyroid carcinoma cells and cerebellar granule neurons, the question arises as to what other cells and tissues might utilize the CaSR/integrin system? In light of the widely distributed expression of the CaSR in many organs and cell types, together with the ubiquitous expression of the integrins, could CaSR/integrin protein complexes function as a universal cell migration/homing complex?
5. In a given tissue or cell type the physiological role of the CaSR may change under pathological conditions. For example, upon transformation from normal to cancerous cells, some evidence suggests that the CaSR is associated with different or opposite effects on cell proliferation vs. metastasis. This phenomenon might be compatible with, or linked to, the evolving concept that rapid cell division and proliferation must be arrested prior to metastasis and tumor cell tissue invasion, and that factors operating to promote either cell division or metastatic tissue invasion might be mutually exclusive (e.g., see Matus et al., 2015). In this context, could stimulating CaSR activity via positive allosteric modulators or calcimimetics hinder tumor cell proliferation, and conversely, could inhibiting CaSR activity via calcilytics or receptor knockdown, block or stymie cancer metastasis? This raises the issue that the development of CaSR therapeutics as applied to oncology could be complicated by the possibility of a CaSR drug promoting tumor cell proliferation while inhibiting metastasis, or vice versa. In any case, a more thorough analysis of CaSR pharmacology, expression levels, and signaling mechanisms in tumor cell biology could ultimately provide solutions that translate into novel therapeutic applications.

## Author contributions

Both authors contributed equally to the text; ST created and prepared the figures.

### Conflict of interest statement

The authors declare that the research was conducted in the absence of any commercial or financial relationships that could be construed as a potential conflict of interest.
